# Direct Modular Printing of Plasmonic Chemosensors

**DOI:** 10.1021/acsami.2c17202

**Published:** 2022-12-14

**Authors:** I. Brian Becerril-Castro, Irene Calderon, Jana Ockova, Matz Liebel, Niek F. van Hulst, Vincenzo Giannini, Ramon A. Alvarez-Puebla

**Affiliations:** † Department of Inorganic and Physical Chemistry, Universitat Rovira i Virgili, Marcel·lí Domingo SN (Edificio N5), 43007 Tarragona, Spain; ‡ ICFO, Av. Carl Friedrich Gauss 3, 08860 Barcelona, Spain; § ICREA, Passeig Lluis Companys 23, 08010 Barcelona, Spain; ∥ Instituto de Estructura de la Materia (IEM), Consejo Superior de Investigaciones Científicas (CSIC), Serrano 121, 28006 Madrid, Spain; ⊥ Technology Innovation Institute, Masdar City 50819, Abu Dhabi, United Arab Emirates; # Centre of Excellence ENSEMBLE3 sp. z o.o., Wolczynska 133, 01-919 Warsaw, Poland

**Keywords:** SERS, wide-field microscopy, chemosensor, nanoparticle inkjet printing, discrete modular sensor

## Abstract

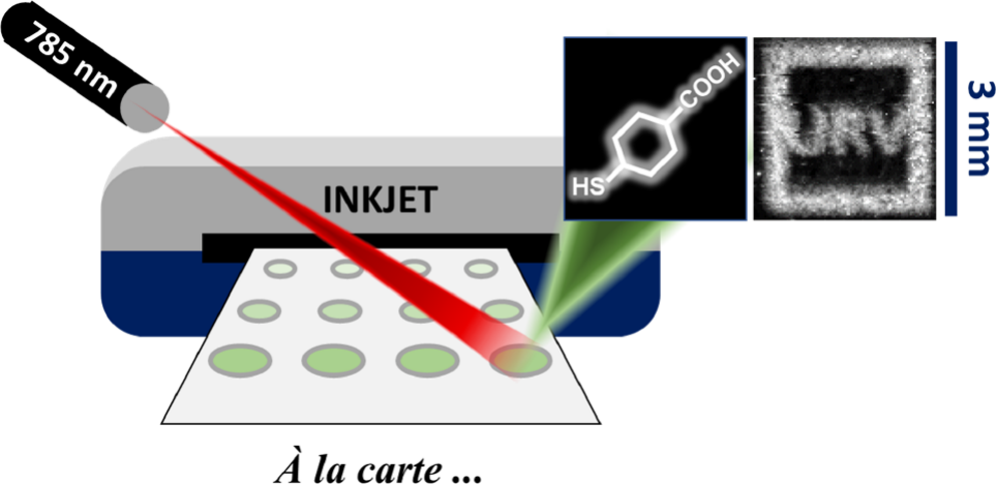

Here, we present and implement a new approach for producing modular inkjet-printable surface-enhanced Raman scattering (SERS) chemosensors. These sensors, combined with a rapid large field-of-view imaging system allow for fast imaging of the chemical characteristics of a sample. The performance of these materials is illustrated by printing a pH sensor on paper and interrogating aqueous solutions at different pH values. Results show single-shot images exceeding 9 mm^2^ which are readily read out via SERS imaging.

## 1 Introduction

Surface-enhanced Raman scattering (SERS) sensors display extremely low limits of detection, down to the single-molecule regime.^1,2^ SERS sensors can be separated into two parts: mechanical support and plasmonic structure. While the mechanical support can be as simple as a glass slide, or a cuvette, up to a complex microfluidic system, the plasmonic part is commonly a nanostructured metallic material that acts as the enhancing element of the Raman scattering signal. Such enhancement stems from the generation of strong electromagnetic fields in the proximity of the surface when illuminated with appropriate light.^3^

The plasmonic materials can be used directly to detect and quantify molecular analytes;^1,2^ however, the exceptional detection limits of SERS, capable of single-molecule detection,^4^ together with the complexity of real samples makes direct detection very difficult.^5^ Additionally, this approach performs poorly for molecules with low cross-section and is incapable of detecting atomic species.^6^ Thus, to detect and/or quantify molecules and atomic ions with SERS in realistic, nonpurified samples, a common methodology relies on so-called chemosensors: molecules with high affinity and selectivity for the target analyte that is functionalized on a plasmonic surface.^7^ Chemosensors exhibit improved sensor specificity and allow multiplexed detection,^8,9^ when multiple particles and analyte-specific molecules are combined. Postacquisition analysis of the SERS spectra, often based on sophisticated statistical methods, then determines the individual contributions.^10,11^ Albeit being complex, this route is often preferred as the production of modular sensors is a cumbersome task. If the nanoparticles (NPs), or metal films, are functionalized a posteriori, it is necessary to prevent the functionalization of the entire plasmonic surface and the cross-contamination among different sensing areas.^12^ If functionalized a priori, a reliable method to deposit the different sensing elements onto one support is needed. For example, the most efficient method for discrete functionalization of several chemosensors within the same surface can be achieved by the deposition of prefunctionalized plasmonic materials in microbeads, mixing of different batches of these microbeads, each batch carrying a different sensor, and depositing them onto an organized surface.^13^ Unfortunately, this method is not clean and the content of each sensor cannot be controlled.^14^ To overcome these drawbacks, deposition alternatives that preserve the optical and chemical nature of the sensors while providing spatial resolution and avoid cross-contamination, are required.

Inkjet printing is a direct deposition manufacturing technique for liquid-phase materials.^15^ Dyes and pigments are the most conventional materials employed to color regular printed pages. It is possible to print metallic NPs into patterns with shapes and sizes that solely depend on the printers’ capabilities on a variety of substrates.^16^

Here, we develop a strategy to produce modular, spatially confined, and organized SERS chemosensors via direct inkjet printing. We then combine these sensors with a single-shot wide-field imaging system^17,18^ to form a chemosensor platform. We show that the SERS response of our plasmonic chemosensor is effectively confined to the ink pattern and, furthermore, possesses good signal-to-noise and signal-to-background ratios, even when imaged over large areas exceeding 9 mm.

## 2 Results and Discussion

Inkjet printing deposits small drops of liquid-phase materials, or ink, onto a substrate. To achieve satisfactory results, a synergy between the printing technology and the ink is required. For piezoelectric printing, the ink formulation must meet specific properties to work within a specific printer. Typical ink properties are viscosities in the range of 1–25 mPa s and surface tensions between 25 and 50 mN/m.^19^ We hence selected a piezoelectric-based printer as this technology offers versatility in the range of inks that can be printed.^20^

**Figure 1:**
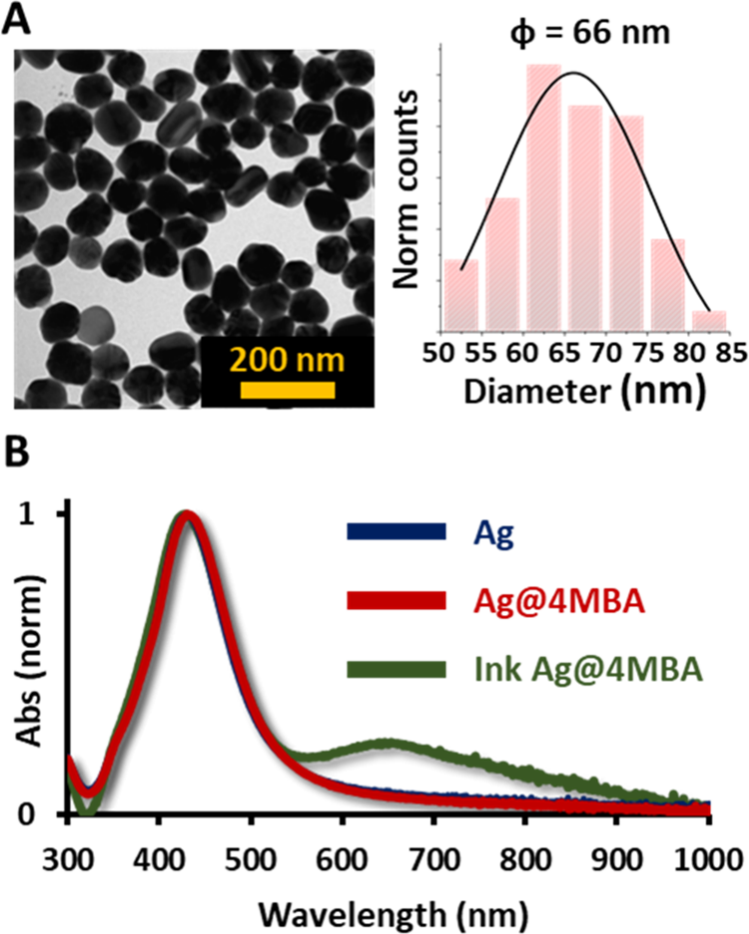
(A) Representative TEM image showing the spherical morphology of the synthesized silver nanoparticles (AgNPs) and their diameter distribution. (B) Experimental surface plasmon resonances of the AgNPs obtained by UV–vis spectroscopy.

For the ink formulation, silver NPs (AgNPs) were selected over gold colloids due to the higher optical activity and wider excitation window of the former.^3^ Spherical particles were preferred over other, more optically active, geometries, such as nanorods or nanostars,^21,22^ as their isometric symmetry allows the formation of three-dimensional hot spots that are aggregation-direction independent.^23^ Finally, citrate-capped NPs were selected because their surface functionalization can be achieved by simple ligand interchange.^24^Figure 1 summarizes characterizations of the 66 nm diameter AgNPs, used in this work, with a maximum localized surface plasmon resonance (LSPR) at 430 nm.

To produce the SERS response in the AgNPs and study their viability as the plasmonic part of the sensors, the AgNPs were functionalized with 4-mercaptobenzoic acid (MBA).^25,26^ Functionalization is achieved by ligand exchange since the Ag-S bond is favorable over the adsorption of citrate. The carboxylic functional group in the opposite direction is likely to maintain stability in the AgNPs after the removal of the adsorbed citrate. Therefore, it is not necessary to employ additional stabilizers to maintain colloidal stability. This rationale was confirmed via ζ-potential measurements yielding −42 mV for the as-prepared citrate AgNPs and −45 mV after MBA functionalization. Finally, the AgNPs were mixed with ethanol and ethylene glycol to produce the plasmonic ink.

Upon ink formulation, a commercial printer and compatible refillable cartridges were used to print different patterns on different types of paper (Figure S2). Note that the NP concentration in the ink is a critical parameter. High colloid concentrations promote uncontrolled aggregation, which may clog the printing nozzles. Too low concentrations result in monodisperse particles on the printing substrate thus not producing sufficient electromagnetic hot spots with the subsequent decrease in the optical efficiency. Thus, as a compromise between these extremes, we concentrate the NPs to create a semistable ink capable of forming a dense collection of hot spots upon printing without clogging the printing nozzle. This ink was repeatedly printed on the desired surface, paper in this case, to investigate the relationship between the nanoparticle density and SERS intensity.

**Figure 2:**
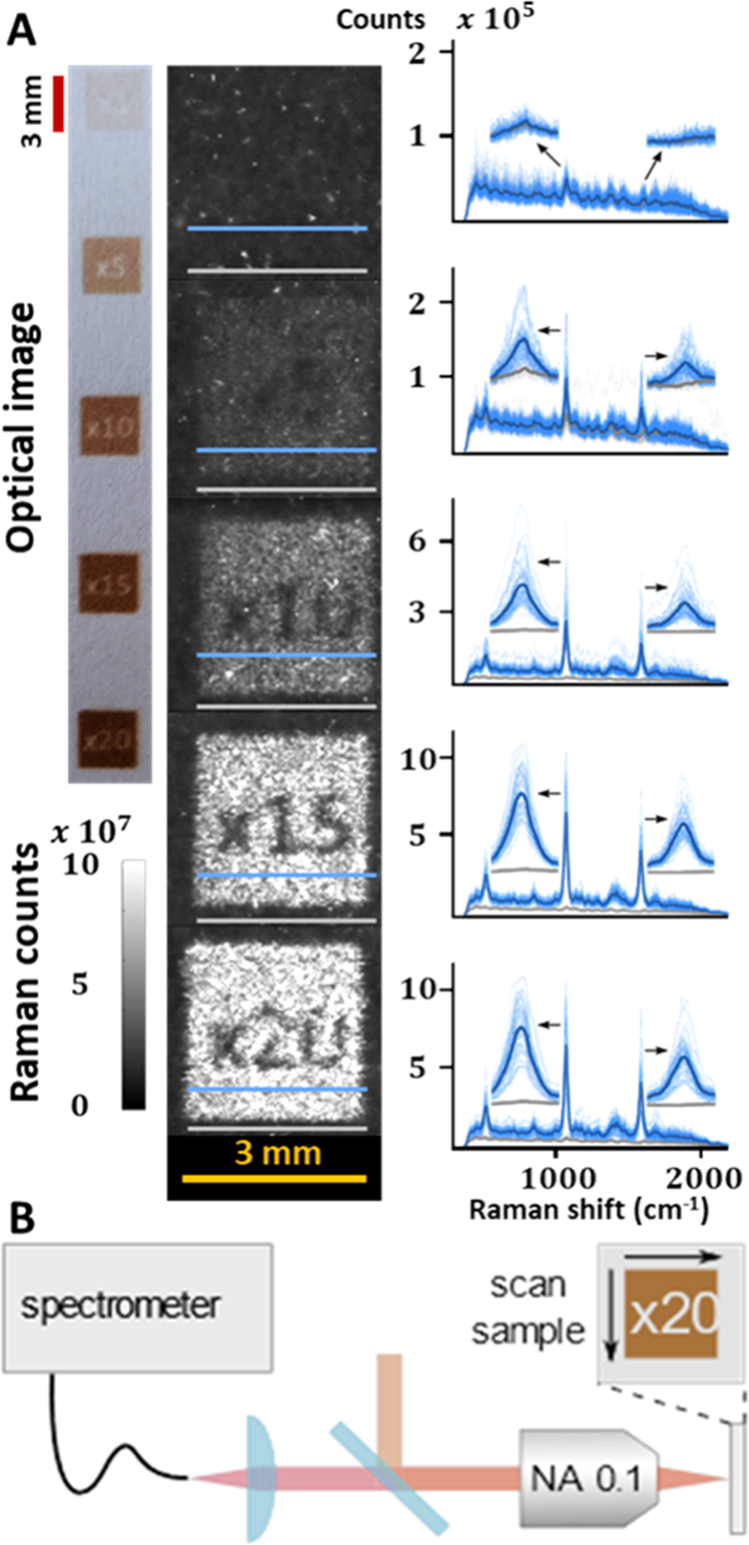
(A) Optical image of the printed patterns, corresponding 2D SERS mappings, and 70 Raman spectra taken along the lines marked. The bold line represents the mean spectrum. (B) Setup diagram of the Raman wide-field spectrometer.

Figure 2A shows an array of five different prints, obtained by printing 1, 5, 10, 15, and 20 times the same pattern, respectively. The expected increase, in contrast, is easily observable by simple optical inspection as the color tends to a darker shade in the optical image. The corresponding SERS two-dimensional (2D) scanning maps were performed over the printed area of 9 mm^2^ in 30 min. We note that 10-fold faster map acquisition is easily possible, by decreasing the waiting time between 2D stage steps. To the best of our knowledge, even the slow proof-of-concept acquisition exhibits an unprecedented signal-to-area collection ratio that, to the best of our knowledge, has never been reported. The SERS images demonstrate that the sensing properties are effectively confined to the printed area. A comparison between spectra acquired along the marked gray and blue lines is shown in Figure 2A, highlighting the signal difference between the unprinted background region and the plasmonic part, respectively. Based on these images, we estimate a minimum printing repetition of five patterns. The SERS signal-to-noise ratio increases with the number of print repetitions. Based on signal-to-background estimates, we conclude that 10–15 repetitions are a good compromise between printing time and SERS signals. Furthermore, excessive repetitions result in a cardboard-like appearance of the paper.

**Figure 3:**
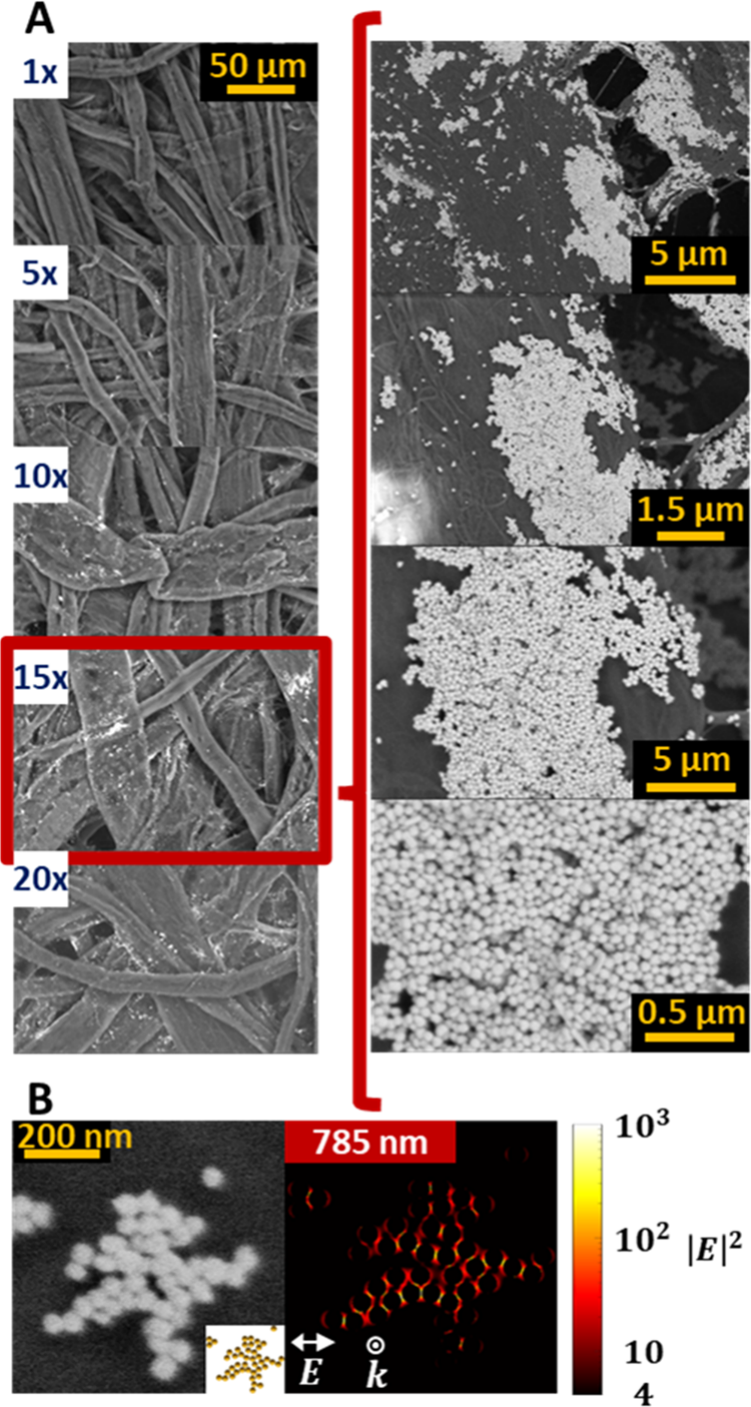
(A) Backscattering SEM images of the multiple printed patterns. Inset: high-resolution images for the 15 times printed material. (B) SEM image and sphere model of a small cluster of AgNPs with correspondent near-field enhancement map. An increase in the local field is produced in the gaps between NPs.

A detailed scanning electron microscope (SEM) characterization of the paper with different numbers of print repeats is shown in Figure 3A. Although a single print aggregate formation is assured, the addition of additional print repeats increases the number of these aggregates and thus the signal. The presence of AgNPs aggregates serves as the complementary explanation for the generation of SERS signal in a material whose main component is not plasmonic but organic. As stated in the literature, clusters of NPs exhibit hot spots in the gaps between them, which increases the intensity of the SERS signal.^27^ To further explain this concept, a small patch of spheres was randomly selected from high-resolution SEM images of the sample corresponding to 15 print repeats to perform a simulation of the optical response using the boundary element method (BEM).^28^Figure 3B shows the near-field enhancement of the packed AgNPs upon 785 nm excitation. We observe the generation of strong electric fields in the interparticle spaces with intensities over 3 orders of magnitude over single particles.

**Figure 4:**
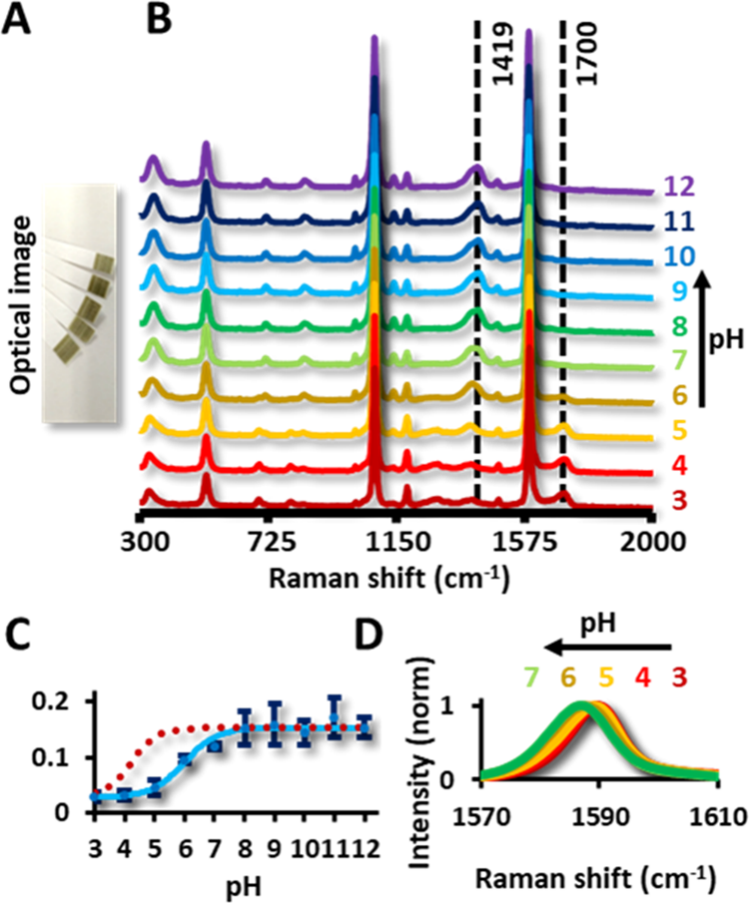
(A) Optical image of the pH sensors and Raman spectra around 1588 cm^–1^. (B) Full Raman spectra (300 to 2000 cm^–1^) in dependence with the pH. (C) Ratio of the intensities at 1700 cm^–1^ and 1419 cm^–1^, corresponding to the COO^–^ and C=O stretch (solid line). Theoretical curve after solving the Henderson–Hasselbach equation (dotted lines). (D) Shift dependence of the ring breathing band with the pH.

To test the optical performance of the fabrication method and the wide-field Raman readout, we used a chemosensor conformed by silver nanoparticles functionalized with mercaptobenzoic acid (NP-MBA), a well-known plasmonic pH sensor.^25^ To this end NP-MBA was directly printed (×15) on paper and cut into the shape of classical pH reactive strips (optical image in Figure 4A). We then immersed these strips into different pH buffer solutions (pH = 3–12). Figure 4B shows the resulting SERS spectra following immersion in the 300–2000 cm^–1^ spectral region. The MBA SERS spectrum is strongly marked by two strong bands about 1590 and 1080 cm^–1^ assigned to the ring breathing and C=C–H in-plane bending, respectively.^29^ Notably, the pH dependence of the spectra is classically depicted as the deprotonation process of the COOH in the MBA molecules, by monitoring the intensity, or area, ratios between the bands at 1419 cm^–1^ (carboxylate stretching, assigned to the COO^–^group) and 1700 cm^–1^ (C=O stretching, corresponding to the COOH group). At a low pH, the COOH group is fully protonated, which causes the spectrum to have a medium-intensity band at 1700 cm^–1^. With increasing pH, deprotonation occurs that increases the concentration of COO^–^ groups, which yields a medium-intensity band at 1419 cm^–1^. One of the pH-sensing methods via MBA-based SERS is to normalize the ν (COO^–^) intensity to the ring breathing mode.^30^Figure 4C shows the ratio between these intensities. The sigmoidal shape is in good agreement with the Henderson–Hasselbach equation for our conjugated acid–base system (dotted line in Figure 4C). A Boltzmann fit gives a p*K*_a_ of 5.06, higher than the predicted value of 4.22, but in good agreement with previous reports as MBA is conjugated to silver rather than free.^31^ Another reported change is the shift of the ring breathing mode with increasing pH. This change is shown in Figure 4D, with a major shift occurring around pH 5, again in good agreement with the previously stated p*K*_a_, Overall, the pH sensing interval is set between 4 and 8, well suited for applications in a mildly acid environment, as often encountered in biological samples. Importantly, our observations highlight that the capabilities of the chemosensor are unaffected by the printing process.

## 3 Conclusions

In summary, we presented and implemented a new approach for producing modular printable SERS sensors, which can be interrogated in a single shot with a wide-field Raman microscope. The approach consists of the use of functionalized NPs with chemosensors and the direct deposition, in the desired pattern, onto a substrate by means of inkjet printing. To illustrate the procedure, we fabricated a pH sensor using MBA as a chemosensor, printed on filter paper. The SERS response is effectively confined to the ink pattern, making this procedure a valid method for producing large-area SERS sensors and images exceeding 9 mm^2^ which are readily read out via SERS imaging. Our results pave the way toward large-scale and inexpensive production of paper-based multisensing arrays with applicability in different scenarios such as environmental science, biology, or medicine.

## 4 Materials and Methods

### 4.1 Materials

All chemical reagents were of analytical grade and used as received unless otherwise noted. All chemicals were obtained from Acros Organics and Sigma-Aldrich. Deionized water (18.2 MΩ·cm^–1^) was used in all reactions. All glassware was cleaned with aqua regia before the experiments.

### 4.2 Characterizations

TEM images were collected from a JEOL 1011. Samples were prepared on carbon-Formvar-coated 200 mesh copper grids. Ultraviolet–visible (UV–vis) spectroscopy was performed in a Thermo Scientific Evolution 201. ζ-potentials were measured in a Zetasizer Ultra from Malvern Panalytical. High-resolution SEM images were taken with a Quanta 600 from FEI company. SERS spectra were acquired in backscattering geometry with a Renishaw inVia Reflex system equipped with a 2D CCD detector, a Leica confocal microscope, and a 785 nm laser line.

### 4.3 2D SERS Imaging

SERS maps were recorded on a custom scanning confocal microscope coupled to a spectrometer. The sample was illuminated with a 781 nm laser through a 0.1 NA air objective underfilled four times to give a spot of ∼15 μm^2^ FWHM and 40 μW μm^–2^ irradiance. The Stokes scattered light was transmitted by a 785 nm long-pass dichroic mirror and a 785 nm notch filter into a custom spectrometer. Each Stokes spectrum was recorded in 50 ms, and each pixel in the 2D SERS map corresponds to the integrated Stokes counts. The sample was scanned with 125 30 μm steps in both *x* and *y* directions.

### 4.4 Synthesis of AgNPs

First, 500 ml of deionized water was heated to boiling point. Next, 6.8 mL of sodium citrate (100 mM), 500 μL of ascorbic acid (100 mM), and a mixture of 1485 μL of silver nitrate (AgNO_3_) (100 mM) with 1118 μL of magnesium sulfate 100 mM were added in sequential order. The mixture of AgNO_3_ and MgSO_4_ was previously incubated for 5 min and added 1 min after the ascorbic acid. The reaction continued under vigorous stirring for 45 min to guarantee total reaction. AgNPs were left undisturbed for cooling.

### 4.5 AgNPs@4MBA

For each 100 mL of the synthesized AgNPs (0.25 mM silver content), 155 μL of 4-MBA (1 mM) was added and left undisturbed for 24 h. The 4-MBA was prepared with ethanol to prevent S–S bonds.

### 4.6 Ink formulation

AgNPs@4MBA were cleaned to eliminate any excess of 4MBA and then concentrated by centrifugation (2500*g*, 20 min). The ink was a mixture of AgNPs, ethanol, and ethylene glycol in a volume ratio 75, 15, and 10%, respectively. The final silver content was 25 mM.

### 4.7 Printing

We use a commercial EPSON-XP2100 and compatible refillable printer cartridges. To clean and avoid accumulation of NPs inside the printer head, mixture volume-to-volume ratios of deionized water, ethanol, and ethylene glycol of 75, 15, and 10% were used as a blank ink. Cleaning of the nozzles was performed if required.

### 4.8 pH Measurements

Fifteen times printed long strips were printed and cut into the shape of classical pH strips. Samples were dipped in the buffer solution and measured over a glass slide for support.

### 4.9 p*K*_a_ Prediction Value

Chemicalize online tool was used for the prediction of the p*K*_a_, ChemAxon (https://www.chemaxon.com). For the simulation, 4-MBA was considered attached to a gold atom through a gold–sulfur bond.

### 4.10 BEM Simulations

To perform the simulation, we employed the boundary element method with the MNPBEM toolbox.^28,32^ The aggregate was depicted as 45 spheres of 66 nm diameter embedded in air (*n* = 1). Every sphere was modeled using 2044 faces, the minimum space between AgNPs was set to 2 nm, and the dielectric constants employed are from Palik’s Handbook.^33^

## Supporting Information Available

Extra transmission electronic microscopy images and comparison of the performance between different papers used as substrates for the sensor imprinting and energy-dispersive X-ray spectroscopy characterization of the films (PDF)

## Abbreviations

SERS: surface-enhanced Raman scattering
NP: nanoparticle
4-MBA: 4-mercaptobenzoic acid
