# Supplementary material for: Direct Modular Printing of Plasmonic Chemosensors
Source: ACS Appl Mater Interfaces. 2022 Dec 14;14(51):57165–70. doi: 10.1021/acsami.2c17202 (PMC9801379; doi:10.1021/acsami.2c17202)
Supplement: Supplementary file 1 — am2c17202_si_001.pdf [file am2c17202_si_001.pdf]

Supporting Information

DIRECT MODULAR PRINTING OF PLASMONIC CHEMOSENSORS

I. Brian Becerril-Castro [a], Irene Calderon [a], Jana Ockova [b], Matz Liebel [b,*], Niek F. van Hulst [b,c,*], Vincenzo Giannini [d,e,f,*], and Ramon A. Alvarez-Puebla [a,c,*]

[a] Department of Inorganic and Physical Chemistry, Universitat Rovira i Virgili, Marcel·lí Domingo SN (Edificio N5), 43007 Tarragona, Spain.

[b] ICFO, Av. Carl Friedrich Gauss 3, 08860 Barcelona, Spain.

[c] ICREA, Passeig Lluis Companys 23, 08010 Barcelona, Spain.

[d] Instituto de Estructura de la Materia (IEM), Consejo Superior de Investigaciones Científicas (CSIC), Serrano 121, 28006 Madrid, Spain.

[e] Technology Innovation Institute, Masdar City, Abu Dhabi, United Arab Emirates.

[f] Centre of Excellence ENSEMBLE3 sp. z o.o., Wolczynska 133, Warsaw, 01-919, Poland.

Corresponding Authors

* Matz Liebel: Matz.Liebel@icfo.eu

* Niek F. van Hulst: Niek.vanHulst@icfo.eu

* Vincenzo Giannini: v.giannini@csic.es

* Ramón A. Alvarez-Puebla: ramon.alvarez@urv.cat

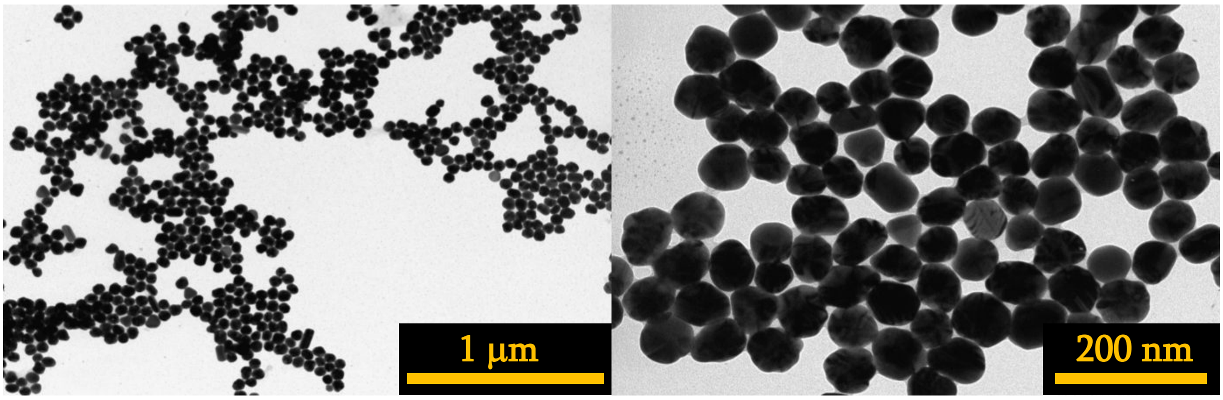

Figure S1. Additional TEM images of the silver nanoparticles used as chemosensors.

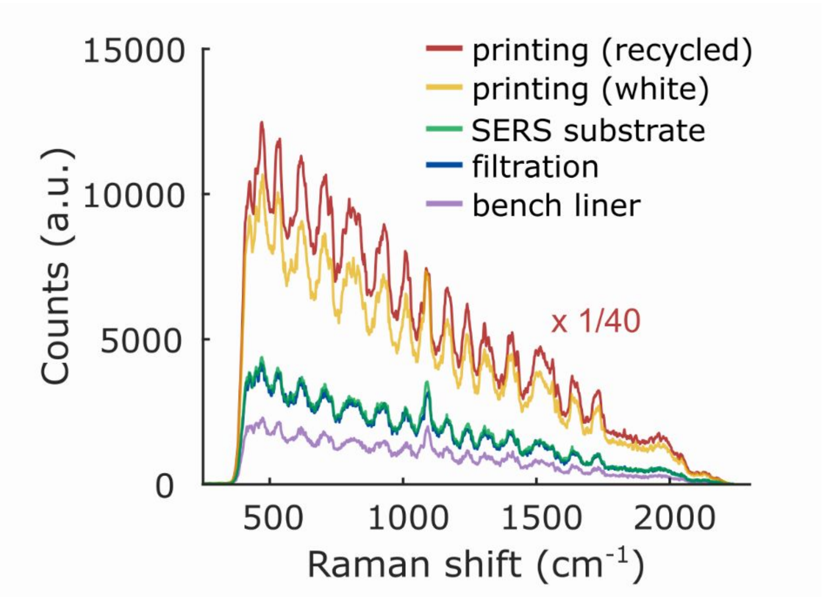

Figure S2. Background signal of different papers. The filtration and bench liner papers were chosen for comparison as chemically inert standards, not directly suitable for printing due to their low density. The white (bleached) paper is the most suitable substrate for Raman illumination at 781 nm. The periodic modulation on the spectra results from etaloning.

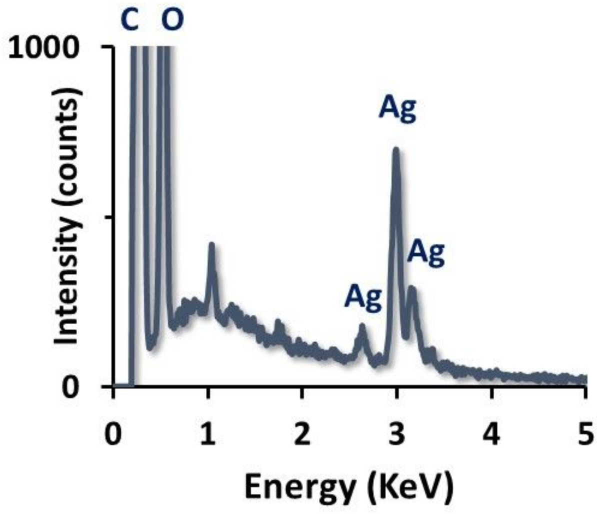

Figure S3. EDX of the sample
